# Persistent Postural-Perceptual Dizziness: A Matter of Higher, Central Dysfunction?

**DOI:** 10.1371/journal.pone.0142468

**Published:** 2015-11-16

**Authors:** Dagny Holle, Benedict Schulte-Steinberg, Sebastian Wurthmann, Steffen Naegel, Ilya Ayzenberg, Hans-Christoph Diener, Zaza Katsarava, Mark Obermann

**Affiliations:** 1 Department of Neurology, University of Duisburg-Essen, Essen, Germany; 2 Department of Neurology, Ruhr University Bochum, Bochum, Germany; 3 Department of Neurology, Evangelical hospital Unna, Unna, Germany; University of Melbourne, AUSTRALIA

## Abstract

**Objective:**

Persistent postural-perceptual dizziness (PPPD) is the most common vestibular disorder in the age group between 30 and 50 years. It is considered to be based on a multisensory maladjustment involving alterations of sensory response pattern including vestibular, visual and motion stimuli. Previous data supported a link between vestibular and pain mechanism. The aim of the study was to investigate whether other sensory inputs such as pain stimuli might be altered in terms of a more widespread central perception dysfunction in this disorder.

**Methods:**

Nociceptive blink reflex was measured in 27 patients with PPPD and compared with 27 healthy, age and gender matched controls. The habituation of the R2 component of the blink reflex was evaluated as the percentage area-under-the curve (AUC) decrease in ten consecutive blocks of five averaged rectified responses. Additionally, clinical characteristics were evaluated.

**Results:**

In patients with PPPD a lack of habituation was observed compared to healthy controls. Relative AUC decreased between the first and the tenth block by 19.48% in PPPD patients and by 31.63% (p = 0.035) in healthy controls. There was no correlation between clinical data (course of disease, comorbid depression, medication, trigger factors) or electrophysiological data (perception threshold, pain threshold, stimulus intensity) and habituation pattern. No trigeminal sensitization in terms of facilitation of absolute values could be detected.

**Conclusion:**

Our study results supports the hypothesis of the multisensory dimension of impaired sensory processing in patients with PPPD extends beyond vestibular/visual motion stimuli and reflexive postural/oculomotor control mechanisms to other sensory inputs such as pain perception in terms of a more generalized disturbed habituation pattern.

## Introduction

Persistent postural-perceptional dizziness (PPPD) is one of the most common causes of chronic dizziness in middle-aged patients and the second most common diagnosis in patients presenting with vestibular symptoms[1] with a high impact on functioning in daily living and quality of life.[2] The main clinical characteristics of this disorder include persisting subjective non-rotational vertigo or dizziness, hypersensitivity to motion stimuli, including the patients own movement and motion of objects in the visual surround, as well as difficulties with precision visual tasks.[3] Typically these patients have normal values in clinical balance tests.[4] Objective tests to prove the diagnosis of PPPD do not exist.

The diagnosis of PPPD is based on a clinical condition named phobic postural vertigo (PPV, Phobischer Schwankschwindel) which was initially described by Brandt and Dieterich in the 1980s. [5] In the early 2000s Staab et al. refined and updated the concept of PPV and renamed it chronic subjective dizziness (CSD). [6] In 2014 new consensus criteria were established and the clinical condition was renamed PPPD. PPPD has now been included by the World Health Organization (WHO) in its draft list to be part of the upcoming International Classification of Diseases (ICD-11) in 2017.

The underlying pathophysiologic mechanisms of PPPD are still enigmatic. PPPD often follows an acute vestibular disorder e.g. vestibular neuritis. Although patients recover from the initial acute disorder and clinical and diagnostics tests become normal again PPPD patients keep on having symptoms of dizziness. Newer concepts, therefore, favor a failure of readapation/habituation of the postural control system in response to acute symptoms of dizziness and vertigo as the main driving force of the disorder. This maladjustment might lead to a misprediction of the sensory consequences of one`s own action explaining the main clinical symptom of dizziness and balance problems in these patients.[4,5] Initially useful high-risk postural control strategies (e.g. moving cautiously when suffering from an acute vestibular neuritis) fail to revert to normal when the initial disorder resolves or is compensated.

Clinical and epidemiological observations as well as previous scientific studies support the concept of an overlap between vestibular and pain mechanisms that might be involved in the pathophysiology of PPPD. On one hand pain disorders such as migraine and balance disorders show a high comorbidity.[7,8] On the other hand migrainous vertigo is the second most frequent cause of recurrent vertigo.[9] Optokinetic stimulation -a primarily vestibular stimulus- intensifies triggered trigeminal pain, and pain sensitivity in the fingers in migraineurs [10] and makes migraineurs more susceptible to develop migraine attacks [11]. Painful trigeminal stimulation itself worsens motion sickness [12] and increases nystagmus in migraineurs.[13] Additive effects of processing afferent vestibular and pain pathways in pre-parabrachial and pre-thalamic areas to the amygadala and cerebral cortex might be responsible for these observations. [14,15] Despite central pathways peripheral mechanism might be additionally involved. For example, similar receptor expressions regarding serotonergic, TRPV1 and purinergic receptors can be found in trigeminal, vestibular and spiral ganglion cells (for review [14]).

The aim of this study was to investigate whether hypothesized maladjusted readaptation/habituation in PPPD extends beyond vestibular/visual motion stimuli and reflexive postural/oculomotor control to other sensory inputs such as pain perception in terms of a more generalized disturbed phenomenon. Therefore, we investigated pain stimulation associated habituation of the R2 component of the nociceptive blink, which reflects short-time adaptation to sensory stimuli,

## Methods

Written and informed consent was obtained from all patients and healthy volunteers before entering the study. The study was performed according the Declaration of Helsinki and the study protocol was approved by the Ethics Committee of the Medical Faculty of the University of Duisburg-Essen, Germany (Approval Number: 13-5482-BO).

### Subjects

Twenty-seven subjects (10 ♂, 17♀) with chronic subjective dizziness according to the diagnostic criteria of Staab et al.[3] (www.who.int/classifications/icd/revision/en/) as well as 27 age- and gender-matched healthy volunteers (HV) were included into the study (Table 1).

**Table 1:** Clinical characteristics of patients with persistent postural-perceptional dizziness.

	Healthy Volunteers(n = 27)	PPPPD atien(n = 27)
**Age (y)**	*39*.*48± 10*.*20*	***39*.*00 ± 8*.*48***
**Gender**	*10 ♂*, *17 ♀*	***10 ♂*, *17 ♀***
**Previous history of migraine**	*0 (0%)*	***1 (4%)***
**Depression**	*0 (0%)*	***4 (15%)***
**Constant dizziness/unsteadiness** *	*n/a*	***27 (100%)***
**Postural relationship** **	*n/a*	***27 (100%)***
**Symptoms since**	*n/a*	
**> 3 month**		***3 (11%)***
**> 1 year**		***10 (37%)***
**2–5 years**		***7 (26%)***
**> 5 years**		***7 (26%)***
**Context-dependent symptom exacerbation** ***	*n/a*	***27 (100%)***
**Triggering factors (Prior neuro-otologic diseases (e.g. benign paroxysmal positional vertigo, vestibular neuritis), Prior medical problems (e.g. mild traumatic brain injury), Prior psychiatric disorders (e.g. anxiety disorder)**	*n/a*	***27 (100%)***
**Normal Physical examination or minor non-diagnostic abnormalities (e.g. scar-related hypesthesia)**	*27 (100%)*	***27 (100%)***
**Normal vestibular laboratory testing** ****	*27 (100%)*	***27 (100%)***
**Normal MRI or minor non-diagnostic abnormalities (e.g. small arachnoidal cyst, not significant white matter lesions)**	*n/a*	***27 (100%)***
**Medication SSRI or SNRI**	***0%***	***4 (15%)***

Values are means ± SD or n (%) of all patients/healthy volunteers

SSRI: Selective Serotonin Reuptake Inhibitor; SNRI: Serotonin-Noradrenalin Reuptake Inhibitor; n/a: not applicable; SD: standard deviation; n: number of patients or healthy volunteers

* Dizziness is present throughout the day but fluctuates in severity

** Symptoms are most severe when walking or standing, less severe when sitting, and absent or very minor when recumbent

*** Provocative factors include active or passive motion that is not related to a specific direction or position, exposure to large-field moving visual stimuli or complex visual stimulation, performance of small-field, precision visual activities such as reading or using a computer

**** Vestibular laboratory testing includes: Videonystagmography (VNG), Vestibular-evoked myogenic potentials (VEMP)

Patients with prior diagnosis of CSD were recruited from the Dizziness and Vertigo Center Essen. They were then re-evaluated to match the new diagnostic criteria of persistent postural-perceptional dizziness (PPPD). All patients fulfilled both diagnostic criteria (CSD and PPPD). PPPD displays the latest consensus for diagnosis of this clinical condition and the World Health Organization has included PPPD in its draft list of diagnoses to be added to the next edition of the International Classification of Diseases (ICD-11) in 2017.[16]

All participants were assessed face to face and demographic data were obtained with a standardised questionnaire including questions from the German version of the Center for Epidemiologic Studies Depression Scale (CES-D) called “Allgemeine Depressionsskala” (ADS). Migraine was diagnosed based on the current diagnostic criteria (ICHD (International Classification of Headache disorders) 3 beta) by an experienced medical doctor. Depression was diagnosed based on the current ICD-10 criteria and ADS by an experienced medical doctor.

One patient suffered from migraine in his childhood but had no migraine attacks after the age of 20. Analysis without this patient did not change study results significantly. Four patients suffered from mild depression. Analysis without these patients did not change study results significantly.

All subjects were instructed not to drink caffeine or alcohol containing beverages within 4 hours before the recording to avoid contamination of study results.

### Electrophysiological settings

The nociceptive blink reflex (nBR) was elicited as described previously.[17,18] Two planar concentric electrodes (inomed Medizintechnik GmbH Lübeck; www.walter-graphtek.com) were attached 10mm above the entry zone of the right supraorbital nerve approximately two cm apart. To avoid unnecessary discomfort for the patients, pain stimulation was restricted to the right side. Stimulation was applied with a triple monopolar square wave pulse lasting 0.5ms. The pulse interval was set at 5 ms, interstimulus interval was 12–18 seconds in a pseudorandomized order. Intensity for stimulation was 2 times individual pain threshold, which was determined with two ascending and descending sequences of successive current intensities between 0.2 and 2 mA in 0.2 mA intensity steps. Additionally, the perception threshold was determined. Surface electrodes recorded rectified EMG-responses over the right orbicularis oculi muscle, referenced to the ipsilateral orbital rim (recording parameters: bandwidth 1Hz to 1 kHz, sampling rate 2.5 kHz, sweep length 300ms (1401 plus, Signal, Cambridge Electronic Design UK)). Ten blocks each of 6 stimuli were applied with an interval of 2 minutes between each block. The 2-min interblock interval was chosen as it has been shown to detect the most reliable habituation in normal subjects.[19] This habituation paradigm has shown efficacy in pervious studies on habituation pattern in migraine patients.[20]

### Data processing

Signal analysis was performed by an investigator blinded to the diagnosis. The first sweep of each block was excluded from further analysis to avoid contamination by the startle response. Area under the curve (AUC) of the R2 response (RA: response area of the R2 response) was analysed in each sweep offline after demeaning, rectification and averaging between 27 and 87ms. Each block was analysed separately, calculating mean values for each block separately. Habituation was defined as percentage change of the AUC between the first and the 10^th^ block of recordings.

### Statistical analysis

**Fig 1:**
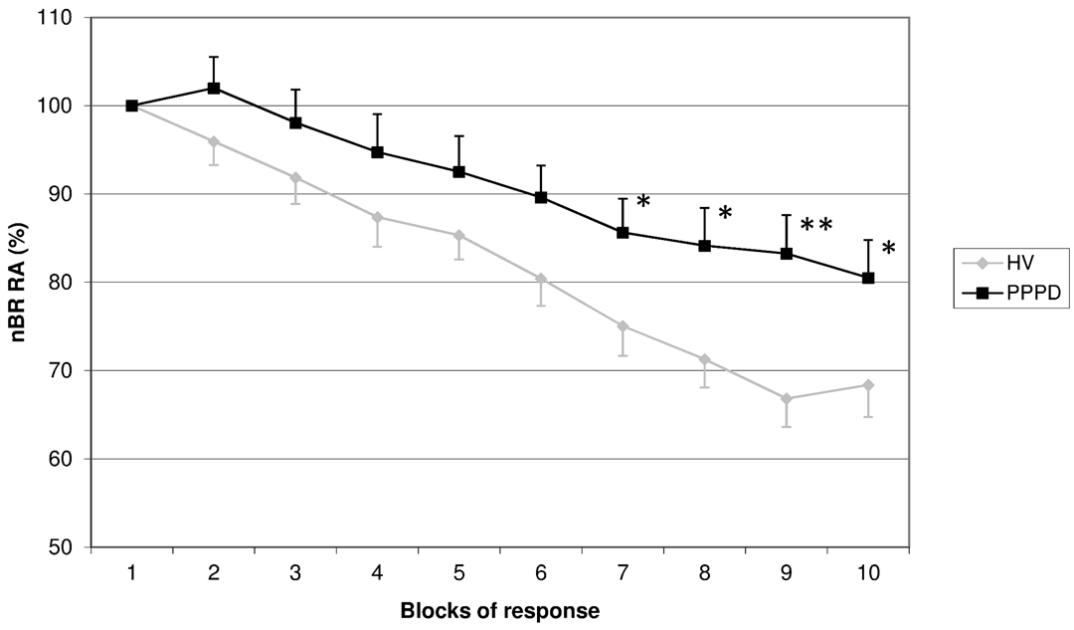
Habituation pattern of the nociceptive blink reflex. Habituation pattern of the nociceptive blink reflex comparing patients with persistent postural-perceptional dizziness (PPPD) and healthy volunteers (HV) showing reduced habituation in patients with PPPD. ANOVA * p < 0.05, ** p <0.005. Habituation of the R2 response area of right-sided nBR in 10 blocks of five averagings. Second to tenth block are expressed as percentage of the first block

One-way analysis of variance (ANOVA) was used to calculate group differences in mean pain threshold, mean stimulus intensities and clinical characteristics. ANOVA for repetitive measurements was applied for measurement of habituation. Therefore, different blocks (1 to 10) were considered as within subject factors and the diagnostic group (HV and PPPD) as between subject factors. Results are presented as means ± standard deviations except in Fig 1 where standard errors were used for better visualization of the different curves. Significance level was set as p<0.05.

## Results

There were no significant differences in mean perception threshold and mean pain threshold as well as mean stimulation intensities (2x pain threshold) comparing PPPD patients and HV (Table 2). We found no significant differences in regard to the R2 latency and area under the curve comparing both groups (Table 2).

**Table 2:** Mean values of electrophysiological findings (block 1).

	Healthy Volunteers	PPPD Patients	Significance level
Perception threshold [mA]	0.33 ± 0.13	0.33 ± 0.19	n.s.
Pain threshold [mA]	0.97 ± 0.52	1.05 ± 0.61	n.s.
Stimulation intensities [mA]	1.67 ± 0.62	1.73 ± 0.55	n.s
AUC [(x10-3)(μV x ms)]	101.19 ± 31.51	125.54 ± 55.42	n.s
N2 latency [ms]	38.90 ± 5.83	40.86 ± 6.05	n.s.

Values are means ± SD

mA: Milliampere; AUC: Area under the curve; μV: Mikrovolt; ms: Milliseconds; n.s.: not significant

PPPD: Persistent postural-perceptional dizziness

Habituation of the nBR RA was significantly reduced in patients with PPPD compared to healthy volunteers (HV) (Fig 1).

In HV a decrease in amplitude of 31.63% between the first and the 10^th^ block of five averagings was detected whereas PPPD patients showed a decrease in amplitude of 19.48%. Habituation gradually increased over the ten blocks in both groups. The difference of the degree of nBR RA habituation between PPPD patients and HV was significant from the 7^th^ to the 10^th^ block of stimulation. No correlation between habituation pattern and other clinical characteristics (course of disease, depression, medication, trigger factors) or electrophysiological results (perception threshold, pain threshold, stimulus intensity) could be detected (data not shown).

## Discussion

Patients with PPPD showed a habituation deficit of the nBR RA of the nociceptive blink reflex suggesting insufficient sensory information processing that goes beyond vestibular system dysfunction supporting the concept of a multisensory maladjustment in this disorder.

This multisensory approach of the underlying pathophysiology of this clinical condition was initially described by Brandt and Dieterich. They postulated in 1986 that PPV (Phobic postural vertigo) is caused by an inadequate compensation of self-induced sensory stimulation.[5] Due to this pathophysiological concept patients predict sensory consequences of their own motoric actions inadequately leading to a disturbed perceptual stability. Clinically, patients feel a reduced balance and gait control with a fear of falling. This subjective impression of balance instability was detected using a pressure sensitive mat (GAITRite^**®**^). Patients with PPV showed an inadequate gait control, which was characterized by a slower walk velocity, reduced cadence and stride length compared to healthy controls.[21] Symptoms even increase when visual control is absent. Furthermore, inappropriate postural strategies involving supraspinal control resulting in increased body sway during normal stance conditions seem also to be involved in this disorder.[22–24] Patients seem to use inappropriate balance strategies during normal stance which are usually used only during difficult and demanding balance tasks, probably due to anxious control of posture.[25,26] Interestingly, during demanding postural tasks such as standing with closed eyes, balance control is comparable to healthy subjects.

Balaban et al. developed in this context the concept of the balance-migraine-anxiety syndrome [15] which might be a suitable pathophysiological framework for PPPD as well and underscores multisensory maladjustment dimension in this clinical condition complex. Based on the clinical observation of increased comorbidity of balance disorders (PPPD/CSD/PPV and neuro-otologic diseases) migraine [27–29] and psychiatric disorders [6,30,31] Balaban et al. hypothesized that additive effects of processing afferent vestibular and pain information in pre-parabrachial and pre-thalamic pathway components to the amygdala and cerebral cortex might be the pathophysiological correlate. [14,15] In line with this hypothesis voxel-based morphometry data showed alterations in vestibular migraine that resemble those of migraine but also extent to areas of the vestibular system.[32] Additionally, functional imagine data of acute migraine attacks also show that the areas of activation also compromise areas of the vestibular central network especially within the dorsal and dorsolateral pons.[33] Despite central effects, peripheral mechanism might also be involved in the overlap of these clinical conditions. Patterns of serotonergic, TRPV1 and purinergic receptor expression are similar in trigeminal, vestibular and spiral (cochlear) ganglion cells.[15] This might be an explanation why similar treatment regimens can be used in this clinical disorders (e.g. antidepressants such as SNRI and SSRI in PPPD. [34,35], migraine [36] and depression[37]; triptans in migraine[38] and motion sickness [39]).

However, only one of our PPPD patients was suffering from migraine in the past Therefore, not all patients seem to display the complete clinical picture of a balance-migraine-anxiety syndrome, although pathways within the underlying pathophysiology of this syndrome might be disturbed. Patients may suffer clinically only from one part of the syndrome e.g. balance disorders, but electrophysiological investigation shows alterations extending beyond vestibular/visual motion stimuli and reflexive postural/oculomotor control to other sensory inputs such as pain perception.

NBR habituation is not a pathognomonic feature of PPPD, but can be observed in other disorders such as migraine. [19,20]. Interestingly, lack of habituation in migraineurs is not restricted to pain modalities, but can also be detected in other sensory modalities such as visually evoked potentials[40], somatosensory evoked potentials (SEP)[41], auditory evoked potentials (AEP)[42] and the vestibulo-collic reflex.[43] It was suggested that this observation reflects an alteration of cortical excitability in general, which goes beyond pure pain processing deficits in migraine. Similar mechanisms might be involved in PPPD as well. Our data support the hypothesis, that higher cortical regions might be involved in the underlying pathophysiology of PPPD, regulating perception of sensory information in a more general way as multiple modalities are involved.

In comparison to other nBR studies, especially to Di Clemente et al, who used the same stimulation paradigm[20], the habituation deficit in PPPD patients seems less pronounced than in pain disorders and can only be detected in the final stimulation blocks (7 to 10). In contrast, in migraineurs, the differences of habituation can be observed as early as the second block of averages. In migraineurs it was shown, that habituation deficit normalizes with increasing attack frequency[20,40]. Our PPPD patients were almost constantly suffering from clinical symptoms of dizziness and balance problems, which might be an explanation why habituation is less pronounced than in migraine patients without headaches. Additionally, one could hypothesize that general central maladjustment only leads as a kind of “side effect” to impaired pain habituation in PPPD compared to the main effect, which would be impaired vestibular processing. In contrast, the main effect in pain disorders such as migraine would strike pain perception and would therefore be more pronounced than in PPPD.

The exact physiological mechanism of habituation and its impairment are unknown, yet. Some authors suggest a lower pre-activation level of the sensory cortex to be a pathophysiological correlate especially in migraineurs, who need a larger activation level after repetitive stimulation which was termed “ceiling effect”.[44] Healthy controls, in contrast, reach the top of response activity and, therefore, habituate faster. Other authors hypothesize that the neurotransmitter dopamine might be involved in the mechanism of habituation. This hypothesis is based on the observation that the habituation deficit which can be detected in Parkinson`s disease is reversible with dopaminergic but not with anticholinergic therapy.[45] Therefore, a dopaminergic deficit has been discussed as pathophysiological correlate of a habituation deficit reflecting sensory disinhibition in this disorder. Animal findings indicate that the dopamine D_1_ receptor plays a role in the generation of habituation to repetitive stimuli whereas D_2_ and D_3_ receptors act to inhibit the habituation process.[46] To which extent a dopaminergic deficit might play a role in PPPD and if affected patients might benefit from dopamine substitution is unknown. In therapy, up to now only SSRIs and SNRIs have been investigated in clinical trials and have shown efficacy in PPPD. [34,35] These substances increase dopamine levels despite their intrinsic serotonergic properties.[47]

Some limitations of this study have to be addressed. We did investigate patients only once. Information on the course of disease, especially regarding a possible regeneration of habituation deficit after the dizziness resolved would be an interesting question. Additionally, analysis of larger subgroups regarding triggering factors, risk factors, medication influences, comorbidities, etc. would be interesting for further analysis as our small patient number per subgroup would probably be to small to detect more subtle differences. Four patients suffered from depression, which might influence habituation. However, exclusion of these patients did not alter the results.

In summary, our study supports the hypothesis of a multisensory dimension of impaired sensory processing in patients with PPPD not only confined to vestibular/visual motion stimuli and reflexive postural/oculomotor control mechanisms but also extended more generally to other sensory inputs and reflexive responses such as pain perception in terms of a disturbed habituation pattern. PPPD might therefore serve as a model supporting the physiological link between vestibular and pain mechanism.

## Supporting Information

S1 File Data set. Raw values of study results of all study participants.
(SAV)
